# Influence of Urethra Sparing on Tumor Control Probability and Normal Tissue Complication Probability in Focal Dose Escalated Hypofractionated Radiotherapy: A Planning Study Based on Histopathology Reference

**DOI:** 10.3389/fonc.2021.652678

**Published:** 2021-05-14

**Authors:** Simon K. B. Spohn, Ilias Sachpazidis, Rolf Wiehle, Benedikt Thomann, August Sigle, Peter Bronsert, Juri Ruf, Matthias Benndorf, Nils H. Nicolay, Tanja Sprave, Anca L. Grosu, Dimos Baltas, Constantinos Zamboglou

**Affiliations:** ^1^ Department of Radiation Oncology, Medical Center – University of Freiburg, Faculty of Medicine, University of Freiburg, Freiburg, Germany; ^2^ German Cancer Consortium (DKTK). Partner Site Freiburg, Freiburg, Germany; ^3^ Berta-Ottenstein-Programme, Faculty of Medicine, University of Freiburg, Freiburg, Germany; ^4^ Division of Medical Physics, Department of Radiation Oncology, Medical Center - University of Freiburg, Faculty of Medicine, University of Freiburg, Freiburg, Germany; ^5^ Department of Urology, Medical Center – University of Freiburg, Faculty of Medicine, University of Freiburg, Freiburg, Germany; ^6^ Institute for Surgical Pathology, Medical Center – University of Freiburg, Faculty of Medicine, University of Freiburg, Freiburg, Germany; ^7^ Department of Nuclear Medicine, Medical Center – University of Freiburg, Faculty of Medicine, University of Freiburg, Freiburg, Germany; ^8^ Department of Radiology, Medical Center – University of Freiburg, Faculty of Medicine, University of Freiburg, Freiburg, Germany

**Keywords:** hypofractionated radiotherapy, PSMA - prostate specific membrane antigen, focal dose escalation, tumor control probability (TCP), NTCP (normal tissue complication probability) model, mpMRI, primary prostate cancer, histopathology

## Abstract

**Purpose:**

Multiparametric magnetic resonance tomography (mpMRI) and prostate specific membrane antigen positron emission tomography (PSMA-PET/CT) are used to guide focal radiotherapy (RT) dose escalation concepts. Besides improvements of treatment effectiveness, maintenance of a good quality of life is essential. Therefore, this planning study investigates whether urethral sparing in moderately hypofractionated RT with focal RT dose escalation influences tumour control probability (TCP) and normal tissue complication probability (NTCP).

**Patients and Methods:**

10 patients with primary prostate cancer (PCa), who underwent 68Ga PSMA-PET/CT and mpMRI followed by radical prostatectomy were enrolled. Intraprostatic tumour volumes (gross tumor volume, GTV) based on both imaging techniques (GTV-MRI and -PET) were contoured manually using validated contouring techniques and GTV-Union was created by summing both. For each patient three IMRT plans were generated with 60 Gy to the whole prostate and a simultaneous integrated boost up to 70 Gy to GTV-Union in 20 fractions by (Plan 1) not respecting and (Plan 2) respecting dose constraints for urethra as well as (Plan 3) respecting dose constraints for planning organ at risk volume for urethra (PRV = urethra + 2mm expansion). NTCP for urethra was calculated applying a Lyman-Kutcher-Burman model. TCP-Histo was calculated based on PCa distribution in co-registered histology (GTV-Histo). Complication free tumour control probability (P+) was calculated. Furthermore, the intrafractional movement was considered.

**Results:**

Median overlap of GTV-Union and PRV-Urethra was 1.6% (IQR 0-7%). Median minimum distance of GTV-Histo to urethra was 3.6 mm (IQR 2 – 7 mm) and of GTV-Union to urethra was 1.8 mm (IQR 0.0 – 5.0 mm). The respective prescription doses and dose constraints were reached in all plans. Urethra-sparing in Plans 2 and 3 reached significantly lower NTCP-Urethra (p = 0.002) without significantly affecting TCP-GTV-Histo (p = p > 0.28), NTCP-Bladder (p > 0.85) or NTCP-Rectum (p = 0.85), resulting in better P+ (p = 0.006). Simulation of intrafractional movement yielded even higher P+ values for Plans 2 and 3 compared to Plan 1.

**Conclusion:**

Urethral sparing may increase the therapeutic ratio and should be implemented in focal RT dose escalation concepts.

## Introduction

Radiotherapy (RT) of primary Prostate cancer (PCa) is currently experiencing an individualization, utilizing modern imaging techniques for staging and definition of intraprostatic gross tumor volume (GTV). Since an increase in RT dose improves tumor control rates (1), concepts of focal dose escalation have developed to deliver higher doses to the tumor and thereby improving rates of biochemical recurrence (2, 3) without risking higher toxicities by respecting OAR restrictions. Recently the long-term result of the phase III FLAME trial demonstrated that mpMRI-defined focal dose escalation significantly improves biochemical disease free survival (4). Earlier publications from this trial demonstrated the feasibility and reported no significant increase in acute and late toxicities (5). These results are encouraging, that unfavorable intermediate- and high-risk PCa patients, who’s proportion is on the rise (6), benefit from these advanced treatments. Besides multiparametric magnetic resonance tomography (mpMRI) being the gold standard for diagnostics in PCa (7), prostate specific membrane antigen positron emission tomography (PSMA-PET) has emerged as a diagnostic tool of high quality (8–13). Recently, the superiority of PSMA-PET for initial staging compared to conventional imaging was prospectively proved, which led to therapy management change in 28% of cases (14). Regarding depiction of the intraprostatic GTV PSMA-PET/CT reveals GTVs more concordant with biopsy reference (9), whereas mpMRI underestimates the true tumor and misses significant tumour lesions (15–17). Previously conducted planning studies from our group and from Goodman et al. demonstrated that despite putative limitations for focal therapy approaches due to larger volumes, boosting of PSMA-PET/CT delineated GTVs is technically feasible (18–20). Bettermann et al. and Eiber et al. could clearly demonstrate that the combined use of mpMRI and PSMA-PET (GTV-Union) significantly improved sensitivity (9, 11). A planning study by Zamboglou et al. revealed significantly increased tissue control probabilities (TCP) for GTV-Union based focal dose escalation compared to GTV-PET or GTV-MRI-based dose escalation (20). Prospective trials will evaluate whether these advances in imaging and diagnostic accuracy can be translated into improved clinical outcomes. A modern approach includes moderately hypofractionated RT (MHRT) to the whole prostate with simultaneously integrated dose escalation to mpMRI- and PSMA-PET/CT-defined GTVs. Although the impact of accountable structures such as bladder, bladder trigone and urethra stay vague, the urethra as a serial organ may be of particular importance in this setting. This planning study aims to investigate whether urethral sparing in MHRT with focal dose escalation delivered to mpMRI and PSMA-PET/CT defined GTVs, influences tumor control probability (TCP) and normal tissue complication probability (NTCP). NTCP was calculated based on the Lyman-Kutcher-Burman (LKB) model with parameters defined by Panettiere et al. (21), TCP was calculated based on 3D dose distribution in co-registered histopathology as standard of reference. Furthermore, the influence of intrafractional movement was assessed (22).

## Material and Methods

### Patient Cohort

The utilized study cohort consisted of ten (10) patients with primary PCa, who underwent 68Ga-HBED-CC-PSMA (68Ga-PSMA-PET) and mpMRI followed by radical prostatectomy. Patient characteristics are listed in **Table 1**. A written informed consent was obtained from each patient and the institutional review board of the Albert-Ludwigs-University of Freiburg approved the study (No.: 469/14).

**Table 1:** Patient characteristics.

Patient	Age (y)	PSA (ng/ml)	TNM	Gleason score
1	67	6.07	pT3a pN1 cM0	3+4 (7a)
2	61	10.57	pT2c pN0 cM0	3+4 (7a)
3	73	25.52	pT2c pN0 cM0	3+4 (7a)
4	59	9.15	pT2c pN0 cM0	4+3 (7b)
5	74	8.82	pT2c pN0 cM0	3+4 (7a)
6	74	15	pT2c pN0 cM0	3+4 (7a)
7	76	20.7	pT2c pN0 cM0	4+3 (7b)
8	73	40	pT3a pN1 cM0	4+5 (9)
9	53	16.3	pT3a pN0 cM0	4+4 (8)
10	72	28.9	pT3b pN1 cM0	4+4 (8)

### PET/CT and MRI Imaging

Diagnostic images were acquired using a diagnostic setup.

PET/CT scans using the ligand 68Ga-HBED-CC-PSMA (23) were performed in 9 patients with a 64-slice GEMINI TF PET/CT and in 1 patient with a Vereos PET/CT (both Philips Healthcare, USA). The imaging systems were cross-calibrated to ensure the comparability of the quantitative measurements and both scanners fulfilled the requirements indicated in the European Association of Nuclear Medicine (EANM) imaging guidelines and obtained EANM Research Ltd. (EARL) accreditation during acquisition. The spatial resolution in the transverse direction near the centre is 4.8 mm for GEMINI TF (24) and 4.2mm for Vereos (25) Patients underwent the whole-body PET scan starting 1 h after injection andwere asked to urinate prior PET imaging. The uptake of 68Ga-PSMA-HBED-CC was quantified by standardized uptake values (SUV). A detailed description of the used 68Ga-HBED-CC-PSMA PET/CT imaging protocol is described in (26).

MR images were acquired on a 3 Tesla system (5 patients on TrioTim, 1 patient on Magnetom Vida, 1 patient on Skyra, all Siemens, Germany) and on a 1.5 Tesla system (3 patients on Aera, Siemens, Germany). The MR imaging systems were equipped with a surface phased array (Body Matrix) in combination with an integrated spine array coil. No endo-rectal coil was used. Not additional cross-calibration was performed. Essentially, T2-weighted fast spin echo (T2W-TSE) images, diffusion weighted images (DWI) and dynamic contrast-enhanced (DCE) perfusion images were acquired. Apparent diffusion coefficient (ADC) maps were calculated from the DWIs using information from all measured b-values. ADC maps were generated with a monoexponential model as implemented in syngo.via (syngo.via ADC & b-value tool, Siemens Healthcare, Germany). Extrapolated high b-value images (b = 1400 s/mm^2^) were calculated with syngo.via using information from all measured b-values. These extrapolated images were considered the high b-value DWIs for prostate MRI reading according to PI-RADS v2.0 (27). MR protocols were heterogeneous in terms of slice thickness, gap between slices and b-values. A detailed description of the used T2w, DWI and DCE MRI imaging protocol can be found in (28).

### Contouring

#### Intraprostatic Tumour Mass

GTV-PET was contoured manually using a validated scaling of SUVmin-max: 0-5 (29) within the prostate using Eclipse™ Treatment Planning System (Varian, USA). GTV-MRI was contoured manually based on MRI T2-w and ADC images, applying imaging criteria PI-RADSv2.0 and considering lesions with a PI-RADS score of ≥ 3 as relevant (27). Final GTVs were the respective consensus contour between two readers with >4 years experience in PET and MRI interpretation. Subsequently careful manual co-registration of *in-vivo* CT and *in-vivo* MRI was performed to transfer GTV-MRI to the corresponding *in-vivo*-CT image and to create GTV-Union composed of the sum of GTV-PET and GTV-MRI. GTV-Union was used based on the benefit in terms of higher sensitivity and complementary information of both techniques (9, 13).

#### Organs at Risk (OAR), Clinical Target Volume (CTV) and Planning Target Volume (PTV)

Bladder, rectum, femoral head as OAR were contoured based on the planning-CT scan according to RTOG guidelines (30). Urethra was contoured based on the co-registered MRI. Planning organ at risk volume (PRV)-urethra was created from applying 2 mm isotropic extension of urethra according to the hypo-Flame trial (31).

**Figure 1:**
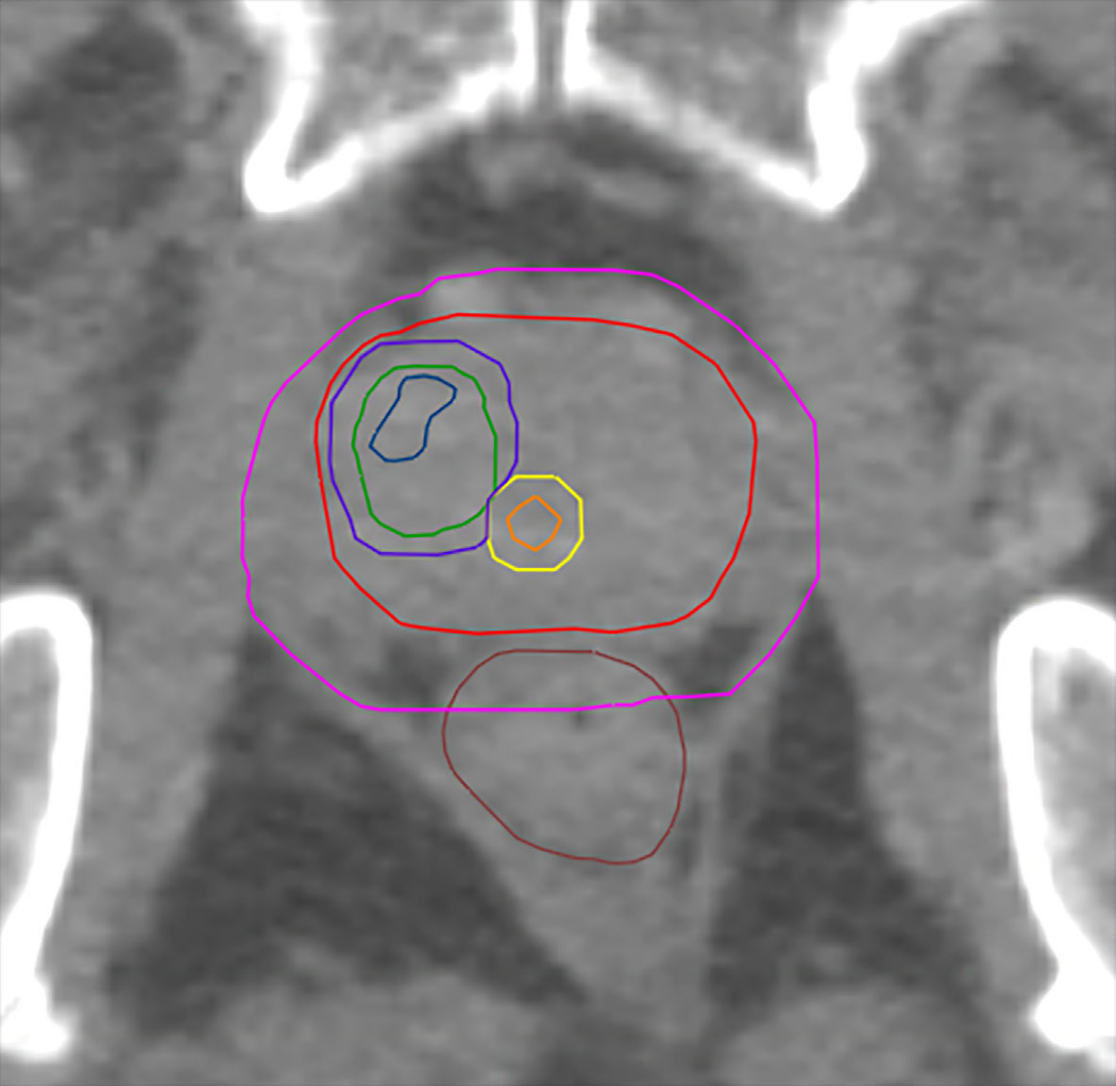
Shows GTV-MRI (blue), GTV-PET (green), urethra (orange) PRV-urethra (yellow), prostate (red), PTV3_3 (boost volume minus PRV-urethra, purple) and PTV1 (pink).

The CTV was created by following the ESTRO-ACROP guidelines (32). CTV1 was defined as the prostate including extracapsular PCa + 3mm isotropic extension (excluding rectum and bladder). CTV_SV was defined as the proximal 1.4 cm and 2.2 cm of the seminal vesicle (SV) in unfavorable intermediate risk and high-risk patients accordingly. In case of tumor infiltration of the SV the respective regions were included in CTV-SV. CTV2 was defined as the prostate and the base of the SV including parts of the SV with visible tumor burden. PTV 1 was created from isotropic 6 mm-extension of CTV1 and 8 mm of CTV_SV, followed by merging both volumes. PTV2 was created from isotropic 6 mm-expansion of CTV2. PTV3 was created from isotropic 2mm-extension of GTV-Union and consequent remove of existing overlaps with organ at risks (OAR) contours. For analysis purposes three different PTV3 were generated: PTV3_1 was defined as the GTV-Union isotropically expanded by 2mm. PTV3_2 was created from the subtraction of urethra from PTV3_1 and finally PTV3_3 was created from the subtraction of PRV-Urethra from PTV_1. See **Figure 1** for illustration of volumes.

### Histopathological Co-Registration

PCa lesions in whole mount histopathology were used as standard of reference as previously conducted by our group (9, 33). After fixation, the resected prostate was fixed in a customized localizer with a 4 mm grid and an *ex-vivo* CT scan (16-channel Brilliance Big Bore, Phillips, Germany) was performed. Subsequently, whole-mount step sections were cut every 4 mm using an in–house cutting device to guarantee equal cutting angles between histological and corresponding *ex-vivo* CT slices. Following paraffin embedding, specimens were cut using a Leica microtome. Haematoxylin and eosin staining were performed following routine protocols. A board-certified experienced pathologist marked PCa lesions. Subsequently, histopathological information was digitalized *via* intermediate registration to *ex-vivo* CT using MITK software (MITK Workbench 2015.5.2). Automatic interpolation was performed to create GTV-Histo (GTV based on histopathology). Images were transferred to Eclipse™ Treatment Planning System v.15.6 (Varian Medical Systems, USA). *Ex-vivo* CT and *in vivo*-CT (from PSMA-PET/CT scans) were carefully manual co-registered allowing non-rigid deformation and considering the 4mm grid and anatomical landmarks such as urethra and cyst and prostate capsule in particular. Hence, this registration workflow takes into account non-linear shrinkage and distortion of the prostate gland after resection.

### Distances to Urethra

Minimum distance of GTV-Histo to urethra was evaluated on each hematoxylin and eosin stained (H&E) slice of the respective patient. Accordingly, minimum distance of GTV-Union to urethra was evaluated on the corresponding CT-slice on the *in vivo* CT.

### IMRT Planning

IMRT plans were created in Eclipse™ Treatment Planning System v15.1 (Varian, USA) with a calculation grid size of 1.5 mm. Dose prescription protocols were the following: PTV1 45 Gy in 15 fractions and PTV2 15 Gy in 5 fractions, resulting in 60 Gy for PTV2. A simultaneous integrated boost (SIB) up to 70 Gy for PTV3 for all 20 fractions was prescribed. Adapted from the DELINEATE trial (34) and based on findings from Martinez et al. (1), our dose concept aimed for boost doses near 100 Gy (EQD2, a/b=1.6). For PTV2 D98% was ≥ 58.8 Gy and D2% ≤ 70 Gy, for PTV3 D98% was ≥ 68.6 Gy and D2% ≤ 71.4 Gy. Three different plans were created using three different boost volumes for the simultaneous integrated boost (SIB): The SIB volumes were PTV3_1, PTV3_2 and PTV3_3 for plan 1, 2 and 3 respectively. Dose constraints for organs at risk were considered according to CHHiP-, FLAME- and DELINEATE-trial (5, 34–36). Dose constraints for Urethra and PRV-Urethra were 62.4 Gy for D2%. Details of RT planning prescription doses and OAR constraints can be found in **Supplementary Material 1**.

To evaluate the impact of urethral sparing three different IMRT plans were calculated: (i) Plan 1 without any dose constraints for urethra, (ii) Plan 2 considered the D2% dose constraint for urethra and (iii) Plan 3 considered the D2% dose constraints for PRV-Urethra.

### TCP and NTCP Modeling

Structure sets and calculated 3D-dose matrices of the radiotherapy plans were exported as DICOM files. Furthermore, using a Varian ESAPI script (https://varianapis.github.io/), dose matrix voxels for each structure were exported (https://github.com/isachpaz/ESAPICommander). TCP was calculated based on the linear quadratic (LQ) Poisson model (37–41):

Eq. 1 $$TCP=\;e^{-\rho^{cl}\cdot V\cdot e^{-\alpha \cdot EQD0}}$$

Where *ρ_cl_* is the homogeneous clonogenic cell density (# cells/cm^3^) in the tumor of volume *V*. *EQD0* is the equi-effective dose for 0Gy fractionation given by Eq. 2, and *α* is the coefficient of LQ-model defining the linear-term of cell killing.

Eq. 2 EQD0=D·(1+d(αβ))

*d* is the dose per fraction, and *D* is the total dose delivered in N fractions, D=N*d, where *α/β* is the *ratio* of linear to quadratic cell killing probability according to the LQ-model.

In the present study, the tumor cell density was set *ρ_cl_*=2.8*10^8^ cells/cm (42–44). *α/β* value of 1.6 Gy was assumed, based on the recent meta-analysis results by Vogelius et al., which included studies with mildly- and ultrahypofractionated radiotherapy (45). To account for diversity of published *α/β* values we performed a robustness analysis for TCP_GTV-Histo_ with three different parameter sets encompassing the range for *α/β* described by Vogelius et al. (45). *α* was each time fitted (**Table 2**), so that 70% TCP_GTV-Histo_ would be reached in our patient cohort with a conventional dose of 60 Gy in 3 Gy fractions, as we have described in our previous publication (19).

**Table 2:** TCP model parameter sets for the robustness analysis.

Parameter set		1	2	3
***ρ* [x 10^8^ cells/cm³]**		2.8	2.8	2.8
***α/β* (5)**		1.2	1.6	2.7
***α* (5)**		0.10099	0.12050	0.15740

NTCP for bladder and rectum (NTCP_Bladder_, NTCP_Rectum_) were calculated based on the relative seriality model as described by Bostel et al. (46). For bladder a *D_50_* of 80.0 Gy as EQD2 for symptomatic contracture and volume loss, a relative seriality parameter value *s* of 1.3 and *γ* = 2.59 were used (47). For rectum a *D_50_* of 80.0 Gy as EQD2 for severe proctitis/necrosis/stenosis/fistula (2, 47–50), *s *= 0.75 and *γ* = 1.79 were considered (47). An *α/β* value of 3.0 Gy for bladder and rectum was assumed (34). For NTCP_Urethra_ the Lyman-Kutcher-Burman (LKB) model was applied for the endpoint urethral stricture as published by Panitierri et al. (21): *D_50_* = 116.7 Gy, *m* = 0.23, *n* = 0.3, and *α/β* = 5.0 Gy. We additionally performed NTCP_Urethra_ calculations for the 68% confident intervals (CI) with a step of 1.0 Gy for D_50_ and a step of 0.01 for *m*. In total 364 combinations of *D_50_* and *m* were evaluated.

### Complication Free Tumour Control Probability P+

In order to account for the injuries or risk for complications to each of the healthy organs (OARs) involved in a given clinical case, the following expression is usually applied for the total probability of injury *P_I_*:

Eq. 3 $$P_{I}=1-\prod_{j=1}^{N_{OARs}}w_{j}\cdot \left(1-NTCP_{j}\right)$$

where *NTCP_j_* is the probability of injuring the normal tissue (OAR), *w_j_* is a weighting factor expressing the relative clinical importance of each endpoint, and *N_OARs_* is the total number of healthy organs involved in the clinical case. The effectiveness of a given dose distribution can be evaluated by the comparison of its advantages in terms of tumour control (benefit *B*) against its disadvantages considering normal tissues complications (injury *I*). The probability of complication free tumour control *P_+_*, is defined as

Eq. 4 $$P_{+}=P(B)-P(B\cap I)=P_{B}-P_{B\cap I}$$

where *P_B_* is the probability of getting benefit from the treatment (tumour control, Eq. 1) and *P_I_* is the probability of causing injury to normal tissues (Eq. 3). For the case of complete independency of response of tumor and OARs, *P_+_* becomes:

Eq. 5 $$P_{+}=P_{B}\cdot (1-P_{I})$$

*P_+_* is an overall parameter for evaluation of complex dose distributions and treatment localisations and is suggested to support decision on treatment plan selection and treatment adaptation (46, 51–56).

### Organ Movement

As previously performed by our group, *TCP* and *NTCP* calculations were calculated with and without movement (22). This was achieved by changing the relative positioning between structure matrix and dose matrix implementing Gaussian filtering of the dose matrix. Based on results of Langen et al. (57) the standard deviation of a three-dimensional Gaussian kernel, was set to 0.92 mm, 1.59 mm and 1.54 mm for left-right, anterior-posterior and cranio-caudal, respectively.

### Statistical Analysis

The Sörensen-dice coefficient was calculated for spatial overlap of GTV-Histo with GTV-Union, GTV-PET and GTV-MRI and for spatial overlap of PTV3_1, PTV3_2, PTV3_3 and GTV-Histo.

Statistical analysis of volumes was performed with GraphPad Prism v8.4.2 (GraphPad Sofware, USA). Data normality was tested using the Shapiro-Wilk test. For not normally distributed variables, Friedman test and uncorrected Dunn’s test was used for comparison of more than two variables and two-sided Wilcoxon matched-pairs signed rank test was used for comparison of two variables (both at a significance level of 0.05). For normally distributed variables, repeated measures one-way ANOVA with the Geisser-Greenhouse correction and Fisher’s LSD was used for comparison of more than two variables and two-sided paired t test was used for comparison of two variables (both at a significance level of 0.05). For statistical analysis of unpaired and not normally distributed data (minimum distance to urethra on H&E slices and CT images) Mann-Whitney test at a significance level of 0.05 was used.

Exploratory statistical analysis of *TCPs*, *NTCPs* and dosimetric analysis was performed with R (version 3.6.2) (58). Wilcoxon matched pairs signed-rank test was used with a significance level of 0.05.

## Results

### Volumes and Distance of GTVs to Urethra

Median volume for GTV-Histo was 4.5 ml (IQR 1.8 – 6.9ml) and for GTV-Union 5.7 ml (IQR 2.9 – 13.3 ml). Median intersection volume of PRV-Urethra with GTV-Histo was 0.05 ml (IQR 0.00 - 0.25 ml) and with GTV-Union 0.1 ml (IQR 0.00 – 0.88 ml) respectively. Expressed in percentage of the PRV-urethra volume, intersection of GTV-Union with PRV-Urethra was median 1.6% (IQR 0.0 – 6.5%) and maximum 8.5% in patient 10. Please see **Supplementary Table 1** for details.

Median volumes for PTV3_1 was 13.5 ml (IQR 7.0 - 22.6 ml), for PTV3_2 13.2 ml (IQR 6.9 – 22.0 ml) and for PTV3_3 12.8 ml (IQR 6.6 – 20.6 ml), respectively. PTV3_3 was not statistically significantly smaller than PTV3_2 (p = 0.053) but significantly smaller than PTV3_1 (p = 0.031) (**Supplementary Table 2**).

The median intersection volume of GTV-Histo with PTV3_1, PTV3_2 and PTV3_3 was 2.7 ml (IQR 1.5 – 6.3), 2.7 ml (IQR 1.5 – 6.2) and 2.7 ml (1.4 – 5.9), respectively. There was no statistically significant difference between the DSCs for GTV-Histo and the three PTVs (p > 0.96) (**Supplementary Tables 2** and **3**).

Median coverage of GTV-Histo by GTV-Union, GTV-PET and GTV-MRI was 79% (IQR 55 – 97%), 76% (IQR 37 – 83%) and 53% (IQR 13 – 74%). Coverage by GTV-Union was significantly higher than by GTV-PET (p = 0.014) and GTV-MRI (p = 0.004), whereas there was no significant difference between GTV-PET and GTV-MRI (p = 0.058). Median coverage of GTV Histo by PTV3_1, PTV3_2 and PTV3_3 was 90% (IQR 70 – 92%), 89% (IQR 70 – 91%) and 85% (IQR 65 – 88%). Coverage by PTV3_3 was significantly lower than by PTV3_1 (p=0.016) (**Supplementary Tables 3** and **4**).

In 3 patients contact between GTV-Histo and urethra could be observed on H&E slices. In 6 patients contact between GTV-Union and urethra could be observed on *in-vivo* CT slices. Discrepancies between patients with detected contact on slices but without intersection volumes were manually verified. In all cases intersection volumes were present but too small to be quantified in Eclipse™ Treatment Planning System. The median minimum distance of GTV-Histo to urethra on each slice was 3.6 mm (IQR 2.2 – 7.3 mm) and median minimum distance of GTV-Union to urethra was 1.8 mm (IQR 0.0 – 5.0 mm). Distance of GTV-Union to urethra was statistically significantly smaller (p = 0.02). Median minimum distance of GTV-Histo to urethra per patient was 1.9 mm (IQR 0.0 – 3.6 mm) and median minimum distance of GTV-Union to urethra per patient was 0.0 mm (IQR 0.0 – 1.5mm). Again, distance of GTV-Union to urethra was statistically significantly smaller (p = 0.02).

### Doses Distribution in Target Volumes

Median D98%, D50% and D2% doses for, PTV3_1-3 (boost volume), GTV-Histo, urethra and PRV-Urethra for plan 1-3 are shown in **Table 3** with and without consideration of the intrafractionary movement, respectively. Without consideration of intrafractionary movement following doses were statistically significant different: For PTV3 D98% of plans 2 and 3 were significantly smaller than for plan 1, whereas D50% showed no significant difference between the three plans. D2% was significantly higher in plans 2 and 3 than in plan 1. For GTV-Histo, only D98% was significantly lower for plan 2 and 3 compared to plan 1. With consideration of intrafractionary movement following doses were statistically significantly different:

**Table 3:** Dose volume parameter values for different volumes without and with consideration of prostate intrafractional movement.

		Without movement			With movement		
		D98%	D50%	D2%	D98%	D50%	D2%
PTV3_1-3*	Plan 1	67.64 (67.27- 67.93)	70.1 (70.08 - 70.1)	71.86 (71.7 - 72.17)	65.76 (65.1 - 66.03)	69.1 (68.72- 69.33)	71.03 (70.69 - 71.14)
	Plan 2	67.0 (66.88 - 67.22)	70.1 (70.07 - 70.15)	72.23 (72.1 - 72.57)	65.77 (65.05 - 65.87)	69.02 (68.69 - 69.23)	71.13 (70.87 - 71.4)
	Plan 3	67.02 (66.9 - 67.25)	70.14 (70.12 - 70.22)	72.25 (72.13 - 72.59)	65.48 (64.96 - 65.74)	68.94 (68.69 - 69.15)	71.31 (70.89 - 71.55)
**GTV-Histo**	Plan 1	68.55 (66.6, 69.02)	70.69 (70.53, 70.84)	72.1 (71.79, 72.76)	67.34 (65.57, 68.01)	70.26 (69.48, 70.34)	71.16 (70.82, 71.35)
	Plan 2	66.35 (65.37, 67.82)	70.73 (70.32, 70.94)	72.51 (72.28, 72.87)	67.05 (65.26, 67.22)	69.92 (69.36, 70.45)	71.42 (70.93, 71.71)
	Plan 3	64.51 (64.01, 66.97)	70.6 (70.31, 70.75)	72.63 (72.4, 72.95)	65.71 (64.26, 66.75)	69.92 (69.08, 70.21)	71.64 (70.91, 71.84)
**Urethra**	Plan 1	59.34 (58.96 - 59.71)	65.95 (62.66 - 66.73)	70.15 (69.66 - 70.69)	57.92 (55.83 - 60.38)	65.45 (62.48 - 66.55)	69.33 (68.74 - 70.27)
	Plan 2	58.27 (57.85 - 58.59)	61.63 (61.34 - 62.4)	66.35 (65.29 - 66.46)	57.79 (56.32 - 59.35)	62.4 (61.57 - 63.65)	66.97 (66.06 - 67.53)
	Plan 3	58.15 (57.49 -58.23)	60.99 (60.54 - 61.58)	64.01 (63.69 - 64.5)	57.64 (56.06 - 59.16)	61.79 (61.22 - 62.51)	65.16 (64.88 - 65.33)
**PRV-Urethra**	Plan 1	58.69 (58.48- 59.41)	65.47 (62.4 - 66.29)	70.55 (70.06 - 71.24)	55.95 (52.34 - 58.79)	64.95 (62.18 - 66.04)	69.72 (69.16 - 70.46)
	Plan 2	58.24 (57.7 - 58.37)	61.81 (61.2 - 62.73)	68.49 (67.54 - 69.17)	55.94 (53.09 - 58.03)	62.37 (61.2 - 63.63)	68.19 (67.16 - 68.54)
	Plan 3	57.92 (57.37 - 58.32)	60.87 (60.69 - 61.85)	66.62 (66.13 - 66.81)	55.9 (52.85 - 57.75)	61.74 (60.87 - 62.61)	66.53 (66.26 - 66.81)

Values in parenthesis represent the observed min-max value range. Dosimetry for PTV3_1-3 (boost volume), GTV-Histo, Urethra, and PRV-Urethra is shown. *Plan 1 is based on PTV3_1, plan 2 on PTV3_2 and plan 3 on PTV3_3.

For PTV3, D98% and D50% of plan 3 were slightly but significantly smaller compared to plan 1, whereas dose parameters of plan 2 showed no statistical significance to dose parameters of plan 3. D2% was significantly higher in plans 2 and 3 than in plan 1. For GTV-Histo D98% was significantly smaller and D2% significantly higher in plans 2 and 3 compared to plan 1.

**Figure 2:**
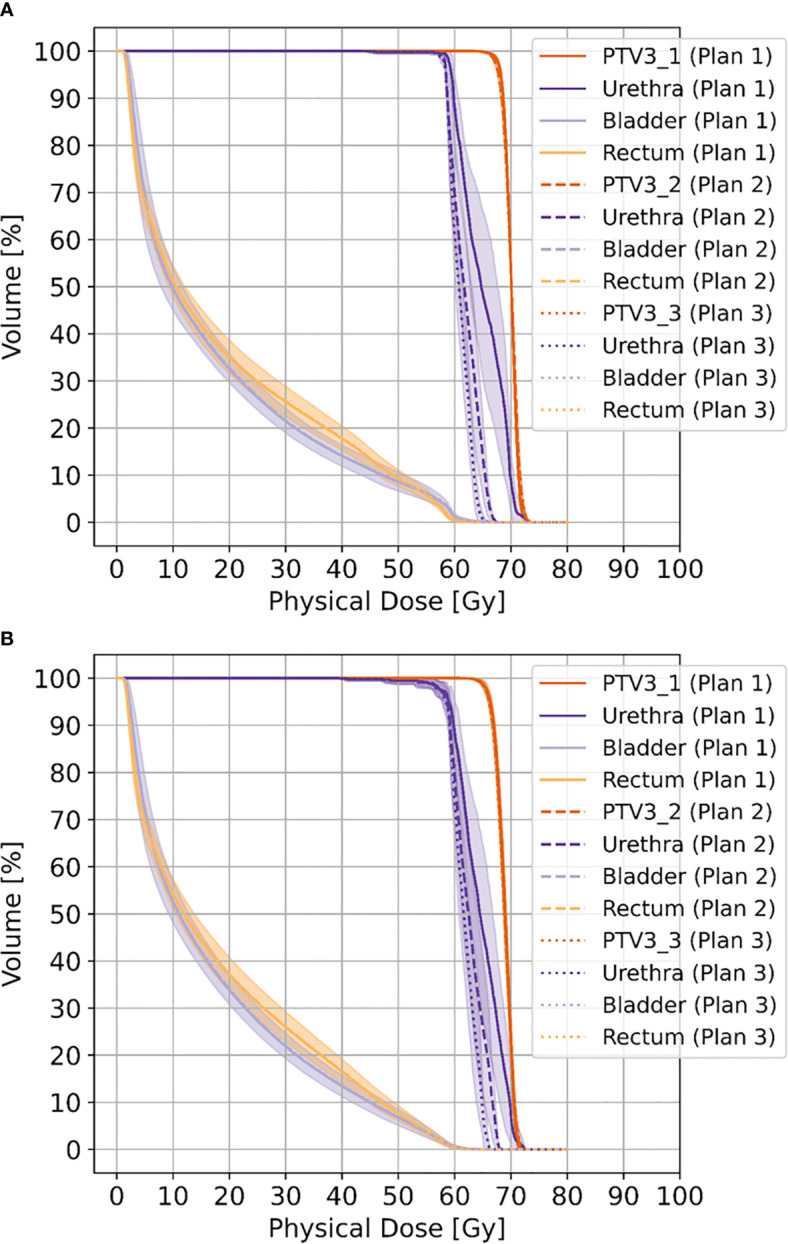
Shows cumulative dose-volume-histograms for boost volumes (PTV3_1-3), urethra, bladder and rectum without movement **(A)** and with movement **(B)**, respectively.

Doses for urethra and PRV-urethra were significantly lower in both plan 2 and plan 3 compared to plan 1 in all cases. Furthermore, all doses were significantly lower in plan 3 compared to plan 2 except for D98% with movement. For details about p-values see **Supplementary Table 6**. **Figure 2** shows cumulative dose-volume-histograms for boost volumes, urethra, bladder and rectum without and with movement.

### Constraints

All plans complied with the constraints for bladder and rectum.

Without consideration of intrafractional movement, in plan 1 (no dose constraints for urethra considered in optimization) constraints for D2% for urethra were not reached in the majority of the planed cases, 8 out of 10. In plan 2 (respecting dose constraints for urethra), constraints for D2% for PRV-urethra were not reached in 7 patients.

When intrafractional movement is considered, in plan 1 constraints for D2% for urethra were not reached again in in 8 patients. In plan 2, constraints for D2% for urethra were not reached in 4 patients and D2% for PRV-urethra in 7 patients.

In plan 3 urethra- and PRV-Urethra constraints were reached in all patients without and with movement consideration.

### TCP/NTCP/P+ Without Intrafractional Movement

Please see **Table 4** for median *P_+_*, *TCP_GTV-Histo_*, *NTCP_Urethra_*, *NTCP_Bladder_* and *NTCP_Rectum_* as well as p-values. Urethra-sparing resulted in significantly lower *NTCP_Urethra_* without significantly affecting *TCP_GTV-Histo_* or *NTCP_Bladder_* and *NTCP_Rectum_*. Consequently, *P_+_* was statically significantly better for plans respecting urethral sparing. Radiobiological modeling was also performed by assuming *α/β* values of 1.2 Gy and 2.7 Gy for tumor tissue (see **Supplementary Tables 7** and **8**). Summarized *P+* shows the same behavior for *α/β* = 1.2 Gy, whereas for *α/β* = 2.7 Gy no significant differences between all three plans could be observed. For the calculation of total probability of injury *P_I_*, required for the complication free tumour control *P_+_* (Eq. 5), all three weighting factors *w_j_* in Eq. 3 for the OARs are set to 1.0 (equal clinical importance). Analysis on patient level revealed, that P+ was higher in plan 2 and plan 3 compared to plan 1 in all patients.

**Table 4:** Median P_+_, TCP- and NTCP-values for plans 1-3, as well as p-values for comparison of plan 1 *vs* 2 and 3, respectively, not considering intrafractional movement with α/β 1.6 Gy for tumor tissue and 3 Gy for bladder and rectum.

	P_+_	TCP_GTV-Histo_	NTCP_Urethra_	NTCP_Bladder_	NTCP_Recutm_
**Plan 1**	0.888	0.997	0.072	0.023	0.009
**Plan 2**	0.919	0.995	0.047	0.022	0.009
**Plan 3**	0.919	0.992	0.042	0.023	0.009
	**p-value**				
**Plan 1 *vs*** **Plan 2**	0.006	0.492	0.002	1.0	0.846
**Plan 1 *vs*** **Plan 3**	0.006	0.275	0.002	0.846	0.846
**Plan 2 *vs*** **Plan 3**	0.922	0.625	0.037	1.0	1.0

The Lyman-Kutcher-Burman model with an α/β of 5 Gy was applied for urethra.

### TCP/NTCP/P+ With Intrafractional Movement

Implementation of intrafractional movement into the model yielded in even slightly higher P+ for both urethral sparring plans (**Table 5**). For median *P_+_*, *TCP_GTV-Histo_*, *NTCP_Urethra_*, *NTCP_Bladder_* and *NTCP_Rectum_* as well as p-values considering intrafractional movement see **Table 5**. Radiobiological modeling was performed by assuming *α/β* values of 1.2 Gy and 2.7 Gy for tumor tissue (see **Supplementary Tables 9** and **10**), and summarized *P_+_* shows again the same behavior for *α/β* = 1.2 Gy, whereas for *α/β* = 2.7 Gy no significant differences between all three plans could be observed. Analysis on patient level revealed, that P+ was higher in plan 2 and plan 3 compared to plan 1 in all patients.

**Table 5:** Median P_+_, TCP- and NTCP-values for plans 1-3, as well as p-values for comparison of plan 1 *vs* 2 and 3, respectively, considering intrafractional movement with α/β 1.6 Gy for tumor tissue and 3 Gy for bladder and rectum.

	P_+_	TCP_GTV-Histo_	NTCP_Urethra_	NTCP_Bladder_	NTCP_Recutm_
**Plan 1**	0.900	0.995	0.069	0.013	0.006
**Plan 2**	0.919	0.994	0.051	0.012	0.006
**Plan 3**	0.923	0.992	0.047	0.012	0.006
	**p-value**				
**Plan 1 *vs* Plan 2**	0.027	0.625	0.006	0.922	1.0
**Plan 1 *vs* Plan 3**	0.020	0.322	0.002	0.846	0.846
**Plan 2 *vs* Plan 3**	1.0	0.625	0.131	1.0	0.846

A Lyman-Kutcher-Burman model with an α/β of 5 Gy was applied for urethra.

Re-run of NTCP_Urethra_, calculation in order to consider uncertainties, showed no deviation from initial outcomes.

## Discussion

In the context of focal escalation, the results of our planning study demonstrate that boosting of PSMA-PET/CT and mpMRI defined GTVs using MHRT is technically feasible and prescription doses as well as dose constraints are achieved even when considering organ movement. Furthermore, urethral sparing achieves significantly lower *NTCPs* for urethral toxicities without affecting *TCPs* and *NTCPs* for bladder and rectum, consequently results in a better therapeutic ratio in terms of *P_+_* and should be implemented in focal RT dose escalation concepts. We discuss the different aspects in the following sections in detail.

Urethra sparing is performed in SBRT and brachytherapy, since higher urethral doses are associated with higher GU toxicities (59, 60). The recently published toxicity reports of the hypoFlame trial suggest that prioritization of OAR constraints yields acceptable toxicities for focal dose escalation using SBRT. At the time of publication, the only published toxicity reports of trials investigating moderately-hyofractionated dose escalation and urethral sparing was the DELINEATE trial, which used MRI-defined boost volumes with dose escalation up to 67 Gy and showed slightly higher, but comparable acute and late GI and GU toxicity rates to dose escalation with conventional fractionation (34). In the cohort receiving focal dose escalated MHRT cumulative grade 2 or worse GU and GI toxicities after 3 years were 22.1% and 14.0%, respectively. The dose regimen chosen in our study utilized a higher prescription dose of 70 Gy for the boost volume defined by validated contouring approaches for GTV definition of PET and mpMRI imaging modalities (29, 61). Not surprisingly, volumes for GTV-Union (median 5.7 ml, IQR 2.9 – 13.3 ml) were significantly larger than GTV-Histo (median 4.5 ml, IQR 1.8 – 6.9 ml, p = 0.01). Consequently, prioritization of maintaining standard toxicity rates is pivotal when boosting larger volumes and therefore we conducted this planning study to evaluate the effect of urethral sparing on *NTCPs* and *TCP* to evaluate its potential in MHRT with focal dose escalation with this novel boost volume definition.

Constraints and prescription doses were achieved for all patients as intended in the respective plans. This also applies when implementing organ movement into the plan evaluation. These results suggest that putative negative consequences in terms of under- or overdosing were not relevant and consequently organ movement did not affect the highly conformal IMRT plans. Furthermore, we confirmed the feasibility of the applied dose and constraint prescription.

To evaluate the impact of urethra-sparing we chose as endpoint for *NTCP*_Urethra_ stricture requiring urethrectomy within 4 years based on the LKB model by Panettiere et al. (21). Based on recently published *α/β* values from Vogelius et al. we used an *α/β* of 1.6 Gy to calculate the *TCP*. Considering the published range for *α/β* values, we performed the same analysis with an *α/β* 1.2 Gy and 2.7 Gy. Application of 1.2 Gy yielded similar results with a significant better *P+* for plan 3 (0.924 and 0.9285 without and with movement, respectively, p = <0.01), whereas application of 2.7 Gy resulted in no significant improvement of *P_+_* (see **Supplementary Tables 7–10**). However, the mentioned meta-analysis suggests that *α/β* of 2.7 Gy is likely to be too high, particularly in a setting of hypofractionation. Therefore, we refer on the results derived from *α/β* of 1.6 in the following. Urethral sparing in IMRT planning significantly reduced the median *NTCP_Urethra_* from 7.2% up to 4.2% (p=0.002) with only minimal and statically not significant reduction of median *TCP* from 99.7% up to 99.2% (p = 0.105). Noteworthy, *NTCP_Bladder_* and *NTCP_Rectum_* were not affected by urethral sparing, precluding the possibility of improving *NTCP_Urethra_* at the costs of other toxicities. Consequently, urethral sparing resulted in significantly better *P_+_* value (88.8% *vs* up to 91.9%, p = 0.006). Considering that the urethra is a serial organ the minimum distance of urethra to PCa is of particular clinical relevance. Evaluation of minimum distances of GTV-Union to urethra was significantly smaller than GTV-Histo and in 60% of patients, contact of GTV-Union with urethra could be determined, supporting the rationale of urethra sparing. The minimal impact on *TCP* can be attributed to the small intersection of PCa tissue and urethra. Even applying a margin to the urethra resulted in intersection of GTV-Histo and PRV-Urethra of median 0.8% and maximum 6.4%, intersection of GTV-Union with PRV-Urethra was median 1.6% and maximum 8.5%. Considering volume analysis subtraction of PRV-Urethra from PTV3 resulted in slightly, but significantly smaller PTV-volumes, as well as slightly but significantly lower coverage of GTV-Histo. Nevertheless, coverage was still very high (85%) and even though doses partly showed significant differences between IMRT plans, the mentioned differences had no significant consequences on *TCP_GTV-Histo_*. Additionally, coverage of GTV-Histo by GTV-Union was statistically significantly higher than by GTV-PET or GTV-MRI, supporting the rationale to implement both imaging modalities in boost volume definition. Overall results of volumetric analysis and TCP/NTCP calculation suggest that boosting of GTV-Union is compatible with sufficient urethra sparing in most cases. A study by Leibovich et al., which found that the mean distance from the urethra to the nearest cancer was 3mm (62). In our study median distance of GTV-Histo to urethra was comparable with 3.6 mm. Even though contact with urethra was detectable in the majority of cases, intersection volumes were very small, supporting the estimation of little consequences of intersection of urethra and GTV.

Comparison of our results with different planning studies is hampered due to lack of data. One other study evaluated *NTCP_Urethra_* and showed extremely high NTCP-values >60% by using *TD50* of 70.7 Gy (63). The results of our study are still higher than clinical reported urethral stricture rates after external beam RT (EBRT) ranging between 2-3%, nevertheless applied doses were lower and these studies did not use focal dose escalation (64–66). Considering this aspect our results represent realistic estimations and should be compared with eagerly awaited long term results of clinical trials investigating focal dose escalated EBRT.

Additionally, we simulated intrafractional organ movement in order to evaluate its consequences on IMRT plans and TCP/NTCP calculation. This implementation had slightly positive effects on *P_+_*. Furthermore, urethral sparing did still significantly reduce *NTCP_Urethra_* (from 7.2% to 4.3%) without significantly affecting *TCP_Histo_*, *NTCP_Bladder_* or *NTCP_Rectum_*. Remarkable *NTCP_Bladder_* and *NTCP_Rectum_* were even slightly better. These results suggest that intrafractional movement potentially influences positively *P_+_* and the used margins were adequate to compensate intrafractional movements. This complies with previously reported results by Thomann et al., which demonstrated this effect in cases where boost volumes do not fully comply with GTV-Histo (22). These results are encouraging that urethral sparing might significantly reduce GU toxicities without significantly affecting tumour control, in particular since all patients in our study benefited from urethral sparing in terms of improved P+-values in plan 2 or 3 compared to plan 1. In order to evaluate the clinical benefit of this approach it should be evaluated in clinical trials. This enables to evaluate, whether specific patient subgroups don’t benefit from this approach. Likely, in patients with high tumour burden or niches with radio-resistant PCa cells (67) surrounding the urethra, sparing might not be an advantage. Whether a threshold in terms of absolute or relative volume of intersection between PRV-urethra and boost-PTV exist, from which on positive effects are reversed, should be evaluated in further studies and larger cohorts. In this context, individual radiosensitivity might be another important aspect, possibly causing a reduced tumour control with urethral sparing in patients with low radiosensitivity and a negligible impact of urethra sparing in patients with high radiosensitivity due to sufficient dose delivery. We estimated an equal radiosensitivity for all patients in our planning study. To the best of our knowledge validated surrogate parameters to determine radiosensitivity are missing and our data don’t allow do draw conclusions in this regard. Future research might enable to consider this aspect. However, urethra sparing offers another tool for individualizing radiotherapy and acknowledging patients’ preferences, for instance a high demand for safety *vs.* tumour control.

Furthermore, adherence to high quality of image acquisition (68), image co-registration (69) as well as accuracy of delineation (29, 70) and radiation delivery (71) is a prerequisite for implementation of this individualized radiotherapy approach. In the context of delineation, progress in diagnostics has to be considered. Regarding PSMA-PET/CT, Fluorine-18-labeled tracers like 18F-PSMA-1007 have been implemented in nuclear medicine practise. Current research shows, that 18F-PSMA-1007 shows very high sensitivities and high specificities (70, 72). Since accurate delineation of boost volumes for focal therapy approaches depends on the applied windowing (73), usage of validated contouring approaches is necessary (13). Whether usage of 18F-PSMA-1007 affects TCP calculation compared to 68Ga-PSMA should be evaluated in future studies.

We acknowledge the limitations of our study. Firstly, it should be mentioned that the NTCP for the urethra was modeled based on a previous publication of Panettieri et al. (21). The analysis was based on 258 which received EBRT and brachytherapy. Thus, without loss of the generality our analysis was based on a parameter-set, which has been modeled with combined treatment. Secondly, we enrolled a relatively small number of patients, which is a result of the elaborate co-registration pathway of the histopathologic specimens. Thirdly, the co-registration between histopathologic 3D-volumes and cross-sectional images bears risks of uncertainty due to non-linear shrinkage of the prostate after prostatectomy and co-registration mismatch susceptibilities. Consequently, coverage of GTV-Histo boost volumes might lack precision. Fourthly, used models for *TCP* and *NTCP* calculation could not be validated with the institutions own experiences, since the follow-up database of patients treated with mildly hypofractionated EBRT was not sufficient. Fifthly, co-registered images were acquired in a diagnostic setup, potentially affecting image registration and dose calculation. Therefore, the included patients, which are part of a larger cohort, were selected in terms of bowel and bladder preparation and positioning enable BRT planning. However, our experiences for image co-registration are in line with a recently published study, demonstrating no significant differences in MRI acquisition in diagnostic and radiotherapy setups (74). Furthermore, different PET/CT and MRI scanners were used. This limitation was considered by cross-calibration of the PET scanners and a reasonable and recommended slice thickness of 3 mm was acquired in all patients.

## Data Availability Statement

The raw data supporting the conclusions of this article will be made available by the authors, without undue reservation.

## Ethics Statement 

The studies involving human participants were reviewed and approved by Institutional review board of the Albert-Ludwigs-University of Freiburg. The patients/participants provided their written informed consent to participate in this study.

## Author Contributions

SS, IS, and CZ contributed to conception and design of the study. RW and BT created IMRT plans. MB, IS, and DB implemented intrafractional movement modeling. SS and IS conducted the statistical analysis. AS was involved in surgery indication and prostatectomy. PB conducted the histopathological processing and marking of tumor lesions. JR was responsible for conduction and reporting of the PSMA-PET/CTs. MB was responsible for conduction and reporting of the mpMRIs. TS, NN, DB, and AG supervised the study. SS and IS wrote the first draft of the manuscript. CZ wrote sections of the manuscript. All authors contributed to the article and approved the submitted version.

## Funding

Ilias Sachpazidis and Constantinos Zamboglou received funding from the “Dekade gegen Krebs” initiative from the BMBF. SS received funding from the EraPERMED Call 2019 (PersoRad, BMBF). Material and publication fee is funded by the EraPERMED Call 2019 (PersoRad, BMBF).

## Conflict of Interest

The authors declare that the research was conducted in the absence of any commercial or financial relationships that could be construed as a potential conflict of interest.
