# Supplementary material for: Influence of Urethra Sparing on Tumor Control Probability and Normal Tissue Complication Probability in Focal Dose Escalated Hypofractionated Radiotherapy: A Planning Study Based on Histopathology Reference
Source: Front Oncol. 2021 May 14;11:652678. doi: 10.3389/fonc.2021.652678 (PMC8160377; doi:10.3389/fonc.2021.652678)
Supplement: Supplementary file 1 [file DataSheet_1.zip › Material 1.DOCX]

**Prescription doses and organ at risk constraints**

**PTV1:** 45 Gy in 3 Gy per fraction, 5 fractions per week

**Dose must be calculated in subtraction volume between PC1-PC3 (name: Sum_PTV1-PTV3)**

D50% (median) ≥ 45 Gy

D98% ≥ 44.1 Gy (minor deviation: up to 40 Gy)

44.1Gy isodose should encompass PTV1/PC1

**PTV2:** 15 Gy in 3 Gy per fraction, 5 fractions per week

**Dose must be calculated in subtraction volume between PTV2-PTV3 (name: Sum_PTV2-PTV3)**

D50% (median) = 60 Gy (range: 59-62 Gy)

D98% ≥ 58.8 Gy (minor deviation: up to 54 Gy)

D2% ≤ 68-70* Gy

95% isodose should encompass PTV2

*depending on the prescription dose for PTV3

**PTV3*:** First, try to plan for 70 Gy in 3.5 Gy per fraction, 5 fractions per week. When this dosage is not applicable (due to not met dose constraints for organs at risk) try to plan 68 Gy in 3.4 Gy per fraction, 5 fractions per week.

Description dose 70 Gy:

D50% (median) = 70 Gy (range: 68-70 Gy)

D98% ≥ 68.6 Gy (minor deviation: up to 66.5 Gy)

D2% ≤ 71.4 Gy (minor deviation: up to 73.5 Gy)

95% isodose should encompass PTV3

Description dose 68 Gy:

D50% (median) = 68 Gy (range: 67-69 Gy)

D98% ≥ 66.6 Gy (minor deviation: up to 64.6 Gy)

D2% ≤ 69.4 Gy (minor deviation: up to 71.4 Gy)

95% isodose should encompass PTV3

* PTV3 is a simultaneous integrated boost delivered in parallel to PTV1/PC and subsequently to PTV2

**Dose constraints for organs at risk**

According to CHHiP-trial, FLAME-trial and DELINEATE-trial:

|  |  | | Optimal | Mandatory |
| --- | --- | --- | --- | --- |
| **Rectum** | Dose for 20 fractions (Gy) | | Max. volume (%) | Max. volume (%) |
|  | 20 | |  | 85 |
|  | 25 | |  | 80 |
|  | 30 | |  | 57 |
|  | 40 | | 38 | 50 |
|  | 50 | | 22 | 35 |
|  | 57 | | 5 | 15 |
|  | 60 | | 0.01 | 3 |
|  | 64 | |  | 1cc |
|  |  | |  |  |
| **Bladder** | Dose for 20 fractions (Gy) | | Max. volume (%) | Max. volume (%) |
|  | 40 | | 38 | 50 |
|  | 50 | | 22 | 35 |
|  | 60 | | 3 | 5 |
|  | 67 | |  | 1cc |
|  |  | |  |  |
| **Femoral head left and right** | Dose for 20 fractions (Gy) | |  | Max. volume (%) |
|  | 41 | |  | 50 |
|  |  | |  |  |
| **Small bowel** | Dose for 20 fractions (Gy) | |  | Max. volume (cc) |
|  | 41 | |  | 17 |
|  | 47 | |  | Dmax |
|  |  | |  |  |
| **Penile bulb** | Dose for 20 fractions (Gy) | | Max. volume (%) | Max. volume (%) |
|  | 41 | | 50 |  |
|  | 49 | | 20 |  |
| **Urethra or PRV_Urethra** | Dose for 20 fractions (Gy) | | Max. volume (%) | Max. volume (%) |
|  | 62.4 | | 2 |  |
|  | 67.3 | |  | 2 |
|  | 70 | |  | 0.1 cc |
|  |  | |  |  |
| **Sigmoid** | Dose for 20 fractions (Gy) | | Max. volume (%) | Max. volume (%) |
|  | 53 | 3cc | | |
