# Supplementary material for: Influence of Urethra Sparing on Tumor Control Probability and Normal Tissue Complication Probability in Focal Dose Escalated Hypofractionated Radiotherapy: A Planning Study Based on Histopathology Reference
Source: Front Oncol. 2021 May 14;11:652678. doi: 10.3389/fonc.2021.652678 (PMC8160377; doi:10.3389/fonc.2021.652678)
Supplement: Supplementary file 1 [file DataSheet_1.zip › Material 2.DOCX]

Supplementary Tables

Table 1 Volumes of GTV-Histo, GTV-Union as well as Intersection volumes of both volumes with urethra and PRV-urethra

|  | **GTV Histo (ml)** | **GTV Union (ml)** | **Intersection GTV-Histo/ Urethra (ml)** | **Intersection GTV-Histo/ PRV-Urethra (ml)** | **Intersection GTV-Union/ Urethra (ml)** | **Intersection GTV-Union/ PRV-Urethra (ml)** | **Intersection GTV-Union/ PRV-Urethra (%)** |
| --- | --- | --- | --- | --- | --- | --- | --- |
| 1 | 4.1 | 4.3 | 0 | 0.2 | 0 | 0.2 | 4.7 |
| 2 | 4.8 | 2.9 | 0 | 0 | 0 | 0 | 0 |
| 3 | 2.6 | 2.8 | 0 | 0 | 0 | 0 | 0 |
| 4 | 1.9 | 5.9 | 0 | 0.1 | 0 | 0 | 0 |
| 5 | 1.5 | 2.2 | 0 | 0 | 0 | 0 | 0 |
| 6 | 0.9 | 5.4 | 0 | 0 | 0 | 0 | 0 |
| 7 | 6.8 | 13.7 | 0 | 0.1 | 0.2 | 0.8 | 5.8 |
| 8 | 7.1 | 13.1 | 0.1 | 0.4 | 0.2 | 1.1 | 8.4 |
| 9 | 6.7 | 13.1 | 0 | 0 | 0.1 | 0.4 | 3.1 |
| 10 | 10.9 | 28.1 | 0.1 | 0.7 | 0.9 | 2.4 | 8.5 |
| **Median** | **4.5** | **5.7** | **0** | **0.05** | **0** | **0.1** | **1.6** |
| **IQR** | **1.8 - 6.9** | **2.9 - 13.3** | **0 - 0.03** | **0 - 0.3** | **0 - 0.2** | **0 - 0.9** | **0 – 6.5** |

Table 2 Volumes of boost planning target volumes (PTV3_1), with subtraction of urethra-volume (PTV3_2) and with subtraction of PRV-urethra volume (PTV3_3). Additionally Intersection volumes with GTV-Histo are shown.

|  | **PTV3_1 (ml)** | **PTV3_2 (ml)** | **PTV3_3 (ml)** | **Intersection PTV3_1/GTV- Histo (ml)** | **Intersection PTV3_2/GTV-Histo (ml)** | **Intersection PTV3_3/GTV-Histo (ml)** |
| --- | --- | --- | --- | --- | --- | --- |
| **1** | 13.5 | 13 | 11.6 | 2.2 | 2.2 | 1.9 |
| **2** | 7.4 | 7.3 | 6.9 | 2.9 | 2.9 | 2.9 |
| **3** | 5.8 | 5.8 | 5.6 | 2.4 | 2.4 | 2.4 |
| **4** | 13.5 | 13.4 | 13.3 | 1.6 | 1.6 | 1.5 |
| **5** | 5.8 | 5.6 | 5.3 | 1.1 | 1.1 | 1.0 |
| **6** | 12.2 | 12.2 | 12.2 | 0.8 | 0.8 | 0.8 |
| **7** | 25.1 | 24.3 | 22.7 | 6.2 | 6.1 | 5.8 |
| **8** | 21.8 | 21.2 | 19.9 | 6.5 | 6.3 | 6.0 |
| **9** | 20.7 | 20.1 | 19.2 | 6.0 | 6.0 | 5.9 |
| **10** | 43.2 | 41.8 | 39.6 | 10.3 | 10.1 | 9.4 |
| **Median** | **13.5** | **13.2** | **12.8** | **2.7** | **2.7** | **2.7** |
| **IQR** | **7.0 - 22.6** | **6.9 - 22.0** | **6.6 - 20.6** | **1.5 - 6.3** | **1.5 - 6.2** | **1.4 - 5.9** |

Table 3 Dice-Sorensen-Coefficient (DSC) of boost planning target volumes (PTV3_1-3) and GTV-Histo as well as coverage of GTV-Histo by PTV3_1-3 are shown.

|  | **DSC PTV3_1/Histo** | **DSC PTV3_2/Histo** | **DSC PTV3_3/Histo** | **Coverage of GTV-Histo by PTV3_1** | **Coverage of GTV-Histo by PTV3_2** | **Coverage of GTV-Histo by PTV3_3** |
| --- | --- | --- | --- | --- | --- | --- |
| **1** | 0.25 | 0.26 | 0.24 | 54 | 54 | 46 |
| **2** | 0.48 | 0.48 | 0.5 | 60 | 6 | 60 |
| **3** | 0.57 | 0.57 | 0.59 | 92 | 92 | 92 |
| **4** | 0.21 | 0.21 | 0.2 | 84 | 84 | 79 |
| **5** | 0.3 | 0.31 | 0.29 | 73 | 73 | 67 |
| **6** | 0.12 | 0.12 | 0.12 | 89 | 89 | 89 |
| **7** | 0.39 | 0.39 | 0.39 | 91 | 9 | 85 |
| **8** | 0.45 | 0.45 | 0.44 | 92 | 89 | 85 |
| **9** | 0.44 | 0.45 | 0.46 | 90 | 90 | 88 |
| **10** | 0.38 | 0.38 | 0.37 | 94 | 93 | 86 |
| **Median** | **0.39** | **0.39** | **0.38** | **89** | **89** | **85** |
| **IQR** | **0.24 - 0.46** | **0.25 - 0.46** | **0.23 - 0.47** | **70 - 92** | **70 - 91** | **65 - 88** |

Table 4 Dice-Sorensen-Coefficient (DSC) of GTV-Union, GTV-PET and GTV-MRI with GTV-Histo as well as coverage of GTV-Histo by GTV-Union, GTV-PET and GTV-MRI are shown.

|  | **DSC GTV-Union/GTV-Histo** | **DSC GTV-PET/GTV-Histo** | **DSC GTV-MRI/GTV-Histo** | **Coverage of GTV-Histo by GTV-Union (%)** | **Coverage of GTV-Histo by GTV-PET (%)** | **Coverage of GTV-Histo by GTV-MRI (%)** |
| --- | --- | --- | --- | --- | --- | --- |
| **1** | 0.14 | 0.11 | 0.08 | 15 | 10 | 5 |
| **2** | 0.49 | 0.47 | 0.08 | 40 | 38 | 4 |
| **3** | 0.67 | 0.64 | 0.61 | 69 | 58 | 58 |
| **4** | 0.38 | 0.36 | 0.30 | 79 | 74 | 21 |
| **5** | 0.49 | 0.37 | 0.47 | 60 | 33 | 47 |
| **6** | 0.25 | 0.26 | 0.27 | 89 | 78 | 67 |
| **7** | 0.53 | 0.55 | 0.2 | 79 | 79 | 15 |
| **8** | 0.65 | 0.67 | 0.63 | 93 | 82 | 86 |
| **9** | 0.60 | 0.60 | 0.63 | 88 | 84 | 81 |
| **10** | 0.54 | 0.54 | 0.68 | 97 | 96 | 72 |
| **Median** | **0.51** | **0.51** | **0.39** | **79** | **76** | **53** |
| **IQR** | **0.35 - 0.61** | **0.34 - 0.61** | **0.17 - 0.63** | **55 - 90** | **37 - 83** | **13 - 74** |

Table 5 show P+ and TCP values for different α/β parameters (1.2 Gy and 2.7 Gy) with and without movement

|  | α/β 1.2 Gy without movement | | α/β 1.2 Gy with movement | | α/β 2.7 Gy without movement | | α/β 2.7 Gy with movement | |
| --- | --- | --- | --- | --- | --- | --- | --- | --- |
|  | **P+** | **TCP_GTV-Histo_** | **P+** | **TCP_GTV-Histo_** | **P+** | **TCP_GTV-Histo_** | **P+** | **TCP_GTV-Histo_** |
| **Plan 1** | 0.8958 | 0.9986 | 0.9083 | 0.9979 | 0.872 | 0.9841 | 0.8807 | 0.9787 |
| **Plan 2** | 0.9204 | 0.9976 | 0.9232 | 0.9973 | 0.8981 | 0.9786 | 0.8964 | 0.9749 |
| **Plan 3** | 0.9242 | 0.9955 | 0.9285 | 0.9962 | 0.8941 | 0.9681 | 0.8984 | 0.9682 |
| **p-value** | **P+** | **TCP_GTV-Histo_** |  |  |  |  |  |  |
| **Plan 1 vs**  **Plan 2** | 0.00195 | 0.49219 | 0.00391 | 0.69531 | 0.10547 | 0.49219 | 0.27539 | 0.69531 |
| **Plan 1 vs**  **Plan 3** | 0.00391 | 0.23242 | 0.00977 | 0.32227 | 0.76953 | 0.23242 | 1.0 | 0.32227 |
| **Plan 2 vs**  **Plan 3** | 1.0 | 0.55664 | 0.43164 | 0.625 | 0.55664 | 0.55664 | 0.69531 | 0.625 |

Table 6: P-values for comparison of doses between plan 1 and plan 2, as well as plan 3, recpetviely.

|  |  | **Without movement** | | | **With movement** | | |
| --- | --- | --- | --- | --- | --- | --- | --- |
| **Structure** | **Plan comparison (p-values)** | **D98%** | **D50%** | **D2%** | **D98%** | **D50%** | **D2%** |
| GTV-Union | Plan 1 vs Plan 2 | 0.004 | 0.275 | 0.275 | 0.027 | 0.049 | 0.049 |
|  | Plan 1 vs Plan 3 | 0.002 | 0.846 | 0.105 | 0.004 | 0.002 | 0.020 |
| GTV-Histo | Plan 1 vs Plan 2 | 0.020 | 0.625 | 0.432 | 0.049 | 0.557 | 0.020 |
|  | Plan 1 vs Plan 3 | 0.004 | 0.193 | 0.193 | 0.006 | 0.064 | 0.027 |
| PC3 | Plan 1 vs Plan 2 | 0.014 | 0.049 | 0.049 | 0.105 | 0.131 | 0.492 |
|  | Plan 1 vs Plan 3 | 0.006 | 0.006 | 0.160 | 0.020 | 0.006 | 0.322 |
| Urethra | Plan 1 vs Plan 2 | 0.006 | 0.002 | 0.002 | 0.037 | 0.002 | 0.002 |
|  | Sum-1b vs. Sum-3 | 0.004 | 0.002 | 0.002 | 0.010 | 0.002 | 0.002 |

Table 7

Median *P+* and *TCP*-values for plans 1-3, as well as p-values for comparison of plan 1 vs 2 and 3, respectively, not considering intrafractional movement movement with *α/β* 1.2 *Gy for tumor tissue.*

|  | **P+** | **TCP_GTV-Histo_** |
| --- | --- | --- |
| **Plan 1** | 0.8958 | 0.9986 |
| **Plan 2** | 0.9204 | 0.9975 |
| **Plan 3** | 0.9242 | 0.9955 |
| **p-value** | **P+** | **TCP_GTV-Histo_** |
| **Plan 1 vs**  **Plan 2** | 0.00195 | 0.49219 |
| **Plan 1 vs**  **Plan 3** | 0.00391 | 0.23242 |
| **Plan 2 vs**  **Plan 3** | 1.0 | 0.55664 |

Table 8

Median *P+* and *TCP*-values for plans 1-3, as well as p-values for comparison of plan 1 vs 2 and 3, respectively, considering intrafractional movement movement with *α/β* 2.7 Gy for tumor tissue.

|  | **P+** | **TCP_GTV-Histo_** |
| --- | --- | --- |
| **Plan 1** | 0.8720 | 0.9841 |
| **Plan 2** | 0.8981 | 0.9786 |
| **Plan 3** | 0.8941 | 0.9681 |
| **p-value** | **P+** | **TCP_GTV-Histo_** |
| **Plan 1 vs**  **Plan 2** | 0.10547 | 0.49219 |
| **Plan 1 vs**  **Plan 3** | 0.76953 | 0.23242 |
| **Plan 2 vs**  **Plan 3** | 0.55664 | 0.55664 |

Table 9

Median *P+* and *TCP*-values for plans 1-3, as well as p-values for comparison of plan 1 vs 2 and 3, respectively, considering intrafractional movement movement with *α/β* 1.2 *Gy for tumor tissue.*

|  | **P+** | **TCP_GTV-Histo_** |
| --- | --- | --- |
| **Plan 1** | 0.9083 | 0.9979 |
| **Plan 2** | 0.9232 | 0.9973 |
| **Plan 3** | 0.9285 | 0.9962 |
| **p-value** | **P+** | **TCP_GTV-Histo_** |
| **Plan 1 vs**  **Plan 2** | 0.00391 | 0.69531 |
| **Plan 1 vs**  **Plan 3** | 0.00977 | 0.32227 |
| **Plan 2 vs**  **Plan 3** | 0.43164 | 0.625 |

Table 10

Median *P+* and *TCP*-values for plans 1-3, as well as p-values for comparison of plan 1 vs 2 and 3, respectively, considering intrafractional movement movement with *α/β* 2.7 Gy for tumor tissue.

|  | **P+** | **TCP_GTV-Histo_** |
| --- | --- | --- |
| **Plan 1** | 0.8807 | 0.9787 |
| **Plan 2** | 0.8964 | 0.9749 |
| **Plan 3** | 0.8964 | 0.9682 |
| **p-value** | **P+** | **TCP_GTV-Histo_** |
| **Plan 1 vs**  **Plan 2** | 0.27539 | 0.69531 |
| **Plan 1 vs**  **Plan 3** | 1.0 | 0.32227 |
| **Plan 2 vs**  **Plan 3** | 0.69531 | 0.625 |
